# Association between gastroesophageal reflux and bronchopulmonary dysplasia in preterm infants: a systematic review and meta-analysis

**DOI:** 10.3389/fnut.2025.1562939

**Published:** 2025-06-24

**Authors:** XinYi Yu, MengKe Sun, Yu Hu

**Affiliations:** Department of Pediatrics, Shengjing Hospital of China Medical University, Shenyang, China

**Keywords:** gastroesophageal reflux, reflux, bronchopulmonary dysplasia, preterm, infants

## Abstract

**Objective:**

Gastroesophageal reflux (GER) has emerged as a potential contributor to lung injury. This meta-analysis aimed to evaluate the association between GER and bronchopulmonary dysplasia (BPD) in preterm infants.

**Methods:**

A systematic literature search was conducted in PubMed, Embase, Web of Science, and Cochrane Library databases up to Oct 19, 2024. Studies assessing the association between BPD and GER in preterm infants were included. Random-effects models was used to calculate pooled risk ratios (RR) with 95% confidence intervals (CIs). Sensitivity analyses and subgroup analyses were performed to assess the robustness of the findings.

**Results:**

Seven studies were included in the meta-analysis. The overall analysis revealed a non-significant association between GER and BPD (RR = 1.35, 95% CI = 0.91–2.01), but significant heterogeneity was observed across the studies (*p* < 0.001, *I*^2^ = 95.2%). The pooled RR ranged from 1.17 (95% CI = 0.79–1.74) to 1.51 (95% CI = 1.02–2.22) with each study omitted. Funnel plot analysis showed noticeable asymmetry, and Egger’s test confirmed potential publication bias (P > |t| = 0.076). Subgroup analysis revealed that GER diagnosed with clinical therapy or ICD-9 codes was significantly associated with BPD (RR = 1.72, 95% CI = 1.52–1.95 and RR = 2.70, 95% CI = 2.48–2.94, respectively). However, GER diagnosed by pH monitoring did not show a statistically significant association with BPD (RR = 0.86, 95% CI = 0.71–1.05).

**Conclusion:**

Preterm infants with clinically diagnosed GER may face an elevated risk of developing BPD. GER diagnosed by pH monitoring was not associated with BPD.

**Systematic Review Registration:**

https://www.crd.york.ac.uk/prospero/.

## Introduction

Bronchopulmonary dysplasia (BPD) is a chronic lung disease that predominantly affects preterm infants, especially those born at very low gestational ages. Despite advances in neonatal care, BPD continues to impact approximately 10% of very premature infants within this population (1). Gastroesophageal reflux (GER) has emerged as a potential contributor to respiratory complications in preterm infants, including BPD (2). GER refers to the retrograde flow of gastric contents into the esophagus and can occur with or without associated symptoms, affecting more than one-third of healthy infants (3, 4). Preterm infants are more susceptible to GER due to prolonged relaxation response and duration of the lower esophageal sphincter (LES) when stimulated by liquids (5). Neonatal gastroesophageal reflux disease (GERD) develops when the reflux of gastric contents causes symptomatic manifestations or subsequent complications. A retrospective cohort study found that the morbidity of corresponding symptoms or complications caused by GER was as high as 10.3% in all preterm infants (6).

GER has been postulated to exacerbate lung injury through mechanisms such as “silent” aspiration or microaspiration of gastric contents into the airways, leading to inflammation, impaired lung development, and subsequent pulmonary dysfunction in preterm infants receiving mechanical ventilation, particularly in those with developing or established BPD (7, 8). The prevalence of GER in BPD was as high as 42.24% in a prospective cohort study, and these infants were more prone to certain late complications (9). Understanding the relationship between BPD and GER is crucial for optimizing the management and outcomes of preterm infants. GER has been reported to significantly increase the risk of BPD, leading to increased economic and medical burdens due to longer hospital stays and higher costs (6). In another prospective observational cohort study, infants with BPD exhibited no significant differences in GER-related clinical characteristics or morbidity when compared with the group without BPD (10).

Previous studies have reported conflicting findings, with some suggesting a significant association between BPD and GER, while others have failed to demonstrate a consistent link. Therefore, a comprehensive evaluation and synthesis of the available evidence through a meta-analysis is warranted to clarify the relationship between BPD and GER in preterm infants.

## Methods

### Search strategy and selection criteria

We have registered this systematic review (CRD 42023440541) in PROSPERO, and the protocol has not been published. The article follows the reporting guideline of Preferred Reporting Items for Systematic Reviews and Meta-analyses (PRISMA 2020 statement) (11).

We conducted a comprehensive literature search on PubMed (Medline), Embase, Web of Science, and the Cochrane Library, covering databases up to Oct 19, 2024. The search strategy employed a combination of medical subject headings (MeSH) terms and free-text terms. No restrictions were imposed on study types. Additionally, we manually reviewed the references of relevant articles and similar publications in PubMed to identify additional pertinent studies. The detailed search strategy is provided in Supplementary Table 1. Duplicate citations were carefully eliminated.

Two independent reviewers (XinYi Yu and MengKe Sun) screened the titles and assessed the full-text of the identified studies. Data related to the study population, exposure to GER, and occurrences of BPD as reported in the articles were collected. In instances where discrepancies arose, the authors resolved them through constructive discussions.

Inclusion Criteria: (1) Studies that reported on the diagnosis of BPD in preterm infants with GER. (2) Studies that included a control group of preterm infants without GER. (3) Studies that provided detailed information with event numbers or a risk estimate along with confidence intervals. Reviews, conference abstracts, and research conducted in languages other than English were excluded.

### Data analysis

#### Data extraction

From each eligible study, we extracted the following data: first author’s name, publication year, study design, sample size, country of study, gestational age of participants, methods used for assessing and diagnosing GER (GERD), definitions and criteria used for diagnosing BPD, event numbers of BPD, reported odds ratios (ORs) and corresponding 95% confidence intervals (CIs) indicating the association between GER and BPD, as well as any potential confounders that were considered or adjusted for during the analysis.

#### Risk of bias assessment

In the studies ultimately included in this review, the diagnosis of GER was predominantly determined using pH monitoring, treatments for GER, or International Classification of Diseases (ICD) codes. Regarding the objectives’ inclusion timeframe, the diagnosis of BPD were largely based on the National Institute of Child Health 2001 consensus.

We employed the Newcastle-Ottawa scale (NOS) scores to assess the quality of the included studies. The scale evaluated the comparability of groups, the characteristics of the study population, and the identification of GER exposure as the three primary criteria for assessing the quality of each observational study. The maximum score is nine points, and as the score increases, so does the quality of the article.

### Statistical analysis

In this meta-analysis, The risk ratios (RR) was used to estimate and measure the association between GER and the risk of developing BPD in preterm infants. We used a random effect model to pool the RR estimates and obtain an overall estimate. And we assessed the heterogeneity using the *I*^2^ statistics. Egger’s test were used to assess small study impacts such as publication bias.

In cases where the *p* value was less than 0.10 or the *I*^2^ exceeded 50%, it indicated the presence of significant heterogeneity. Sensitivity analysis was conducted to explore the influence of individual studies on the overall pooled result. Additionally, we conducted subgroup analyses based on the different methods of GER diagnosis to investigate the possible sources of heterogeneity.

Statistical significance was defined as a two-tailed *p* value less than 0.05. All statistical analyses were performed using Stata 17.0.

## Results

### Literature search results

Initially, a total of 593 records were obtained from the electronic databases (Supplementary Table 1). According to the PRISMA flowchart, after eliminating 163 duplicate records, 430 records remained for title and abstract screening. Additionally, 91 reviews, 3 abstracts, 316 irrelevant records and 1 study written in French were excluded. Subsequently, we carefully evaluated the full texts of the remaining 19 studies once more. From these, 2 studies were excluded due to the lack of a definite diagnosis for GER (12, 13). Among them, 6 studies were excluded as they did not provide available data on GER (14–19), 3 studies were excluded due to the absence of a non-BPD group (9, 20, 21), and 1 study was excluded because it did not focus on the diagnosis of neonatal BPD (22).

**Figure 1:**
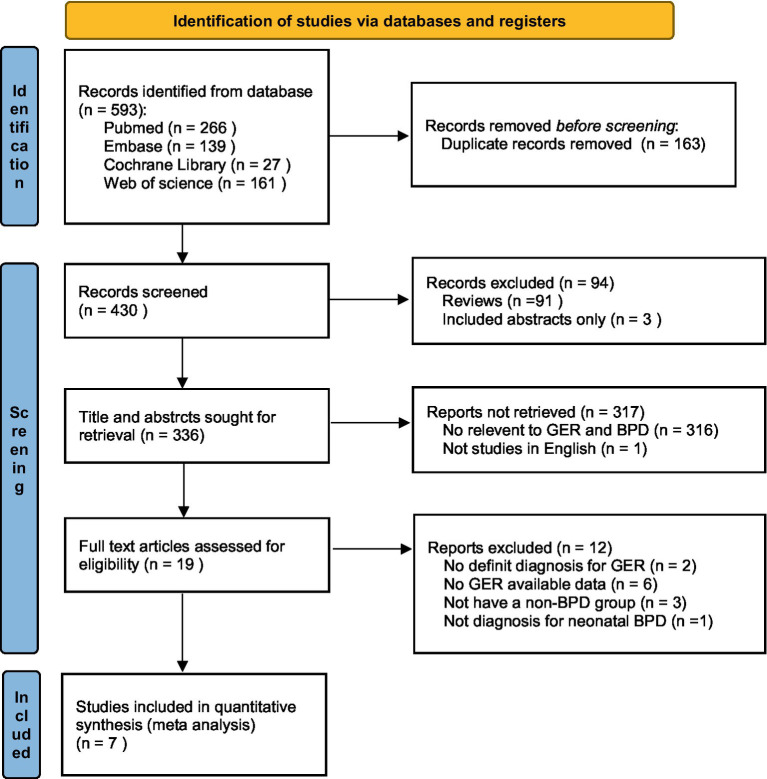
Flowchart illustrating the study selection process for inclusion in this meta-analysis. GER, gastroesophageal reflux; BPD, bronchopulmonary dysplasia.

After meticulous inspection of the articles, a total of seven full-text screening studies were ultimately included in our meta-analysis (6, 10, 23–27). Please refer to Figure 1 for a visual representation of the selection process.

### Study characteristics and quality assessment

As shown in Table 1, six studies were conducted in North America, while one study was conducted in Europe. A total of 19,823 infants were included in the seven studies, out of which 2,359 developed GER. The diagnostic criteria for GER (GERD), as well as the inclusion and exclusion criteria for the study population, are provided in Supplementary Table 2. For BPD diagnosis, all seven studies adopted the criterion of supplemental oxygen requirement at either 28 postnatal days or 36 weeks’ postmenstrual age. Data on cases in each group were collected from all seven articles. Three articles reported OR values along with 95% CIs, but only one study considered confounders and adjusted for birth weight and postconceptional age at the time of pH study.

**Table 1:** Characteristics and details of the included studies.

Study	Year	Study region	Study type	Participant characteristics	GER/GERD diagnosis criteria	NOS scores
Akinola (10)	2004	U.S.	Cohort study	preterm infants (GA < 32 w) *n* = 137 (GER:87; non-GER:50)	pH monitoring RI ≥ 10	7
Frakaloss (23)	1998	U.S.	Cohort study	preterm infants (GA ≤ 37 w) *n* = 46 (GER:23; non-GER:23)	pH monitoring (RI > 5), milk scan, and barium contrast study	7
Fuloria (24)	2000	U.S.	Cohort study	preterm infants (BW ≤ 1,500) *n* = 719 (GER:160; non-GER:559)	Tests or treatment for GER	7
Jadcherla (6)	2013	U. S.	Cohort study	preterm infants (GA ≤ 36 w) *n* = 18,567 (GER: 1907; non-GER:16660)	International Classification of Diseases, Ninth Revision (ICD-9) code of 530.81.	8
Khalaf (25)	2001	U.S.	Cohort study	All NICU infants *n* = 150 (GER:84; non-GER:66)	pH monitoring RI ≥ 6	7
Manti (26)	2020	Italy	Pilot cohort study	preterm infants (GA < 32 w) *n* = 30 (GER:11; non-GER:19)	Reflux with pathologic consequences requiring any kind of antireflux therapy	7
Mezzacappa (27)	2008	U.S.	Cohort study	Preterm infants (BW < 2,000 g, GA ≤ 37 w) *n* = 174 (GER:87; non-GER:87)	pH monitoring RI ≥ 10	8

GER, gastroesophageal reflux; GERD, gastroesophageal reflux disease; NOS, Newcastle–Ottawa scale; GA, gestational age; BW, birth weight; RI, reflux index.

The risk of bias in the studies was assessed using the NOS scores (Supplementary Table 3). Each study received a total score ranging from 7 to 8, indicating relatively good overall study quality.

### Risk of BPD in preterm infants with GER

**Figure 2:**
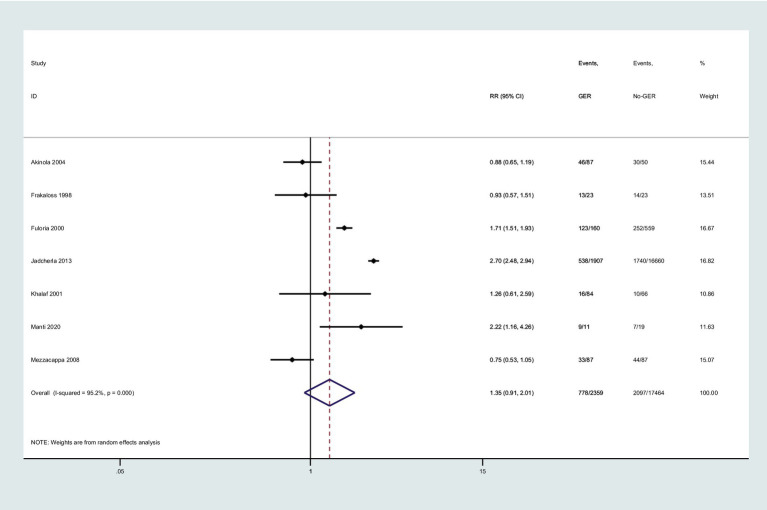
Forest plot displaying the relationship between GER and the risk of BPD (RR = 1.35, 95% CI = 0.91–2.01, *I*^2^ = 95.2%, *p* < 0.001). The analysis employed a random-effect model. Gray boxes represent study estimates with sizes corresponding to analytical weights. Lines within boxes depict the 95% CIs of each study. The pooled estimate is depicted as a vertical dashed black line, with its 95% CI shown as a diamond. GER, gastroesophageal reflux; BPD, bronchopulmonary dysplasia; RR, risk ratio; CI, confidence interval.

Seven studies investigated the association between GER and BPD in preterm infants. Utilizing a random-effects model, we pooled the effect estimates. The forest plot demonstrated an increase in the risk of developing subsequent BPD in preterm infants with GER, but this relationship did not reach statistical significance (RR = 1.35, 95% CI = 0.91, 2.01, Figure 2). Moreover, substantial heterogeneity was observed across the studies (*p* < 0.001, *I*^2^ = 95.2%).

### Sensitivity analysis and publication bias

Regarding sensitivity analyses, we also performed a fixed-effect model to pool the effect estimate, and we observed significant statistical significance (RR = 2.17, 95% CI = 2.03, 2.32, Figure 2). This indicates that the presence of small-sample studies contributed to the observed heterogeneity, which had a substantial impact on the combined effect size. Furthermore, we conducted Egger’s tests to quantitatively evaluate publication bias, and the risk of GER was found to be affected by small-study effects (P > |t| = 0.076 for Egger’s test).

**Figure 3:**
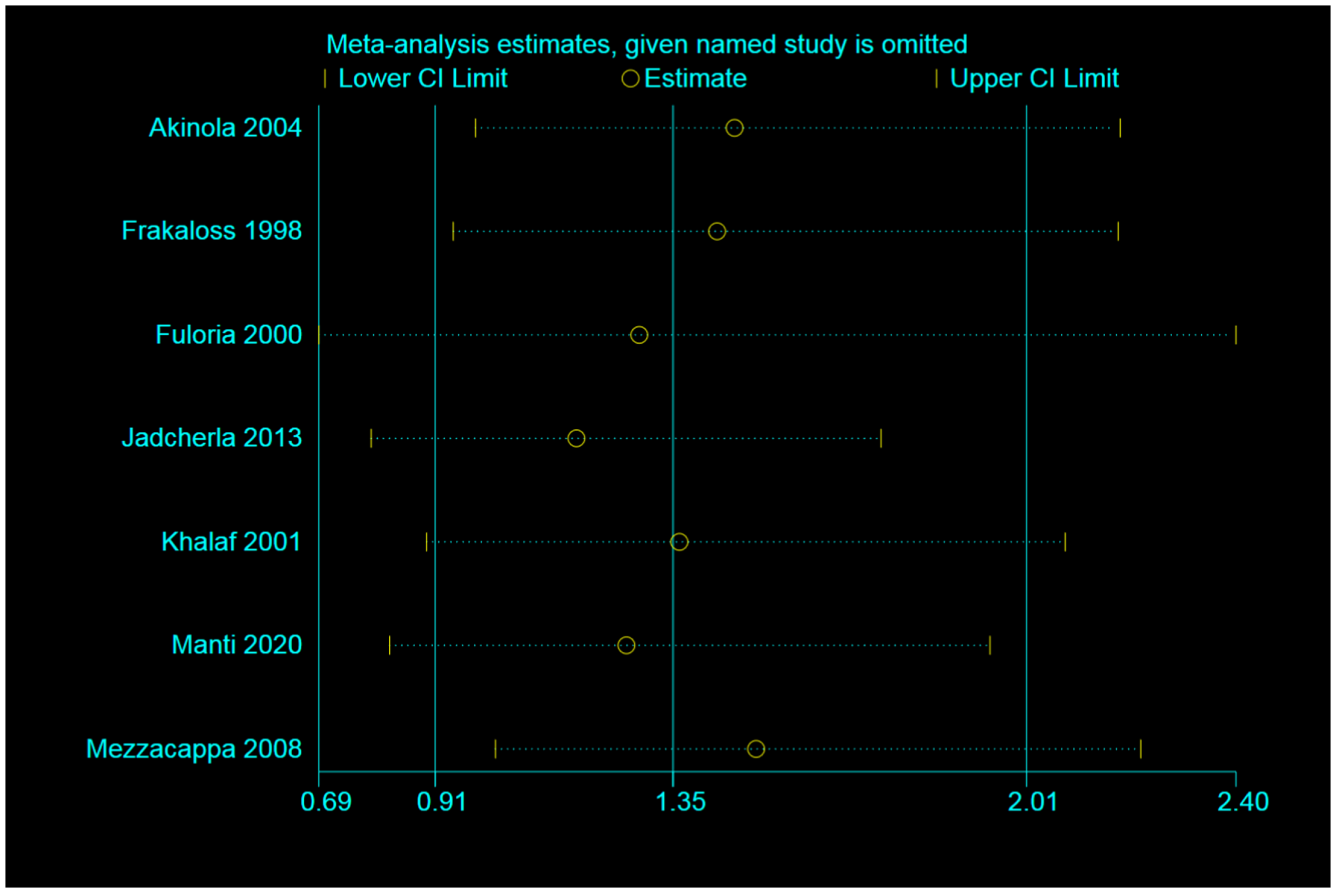
Sensitivity analysis illustrating the effect of omitting individual studies on random-effect estimates (exponential form).

As depicted in Figure 3, the effect of individual studies on the overall study was found to be small. In the sensitivity analyses, we systematically eliminated one study at a time, recalculating the pooled RRs of the remaining studies. After excluding the studies by Jadcherla et al. and Mezzacappa et al. (6, 27), the pooled RRs did not undergo significant changes and ranged from 1.17 (95% CI = 0.79–1.74) to 1.51 (95% CI = 1.02–2.22, Figure 3).

**Figure 4:**
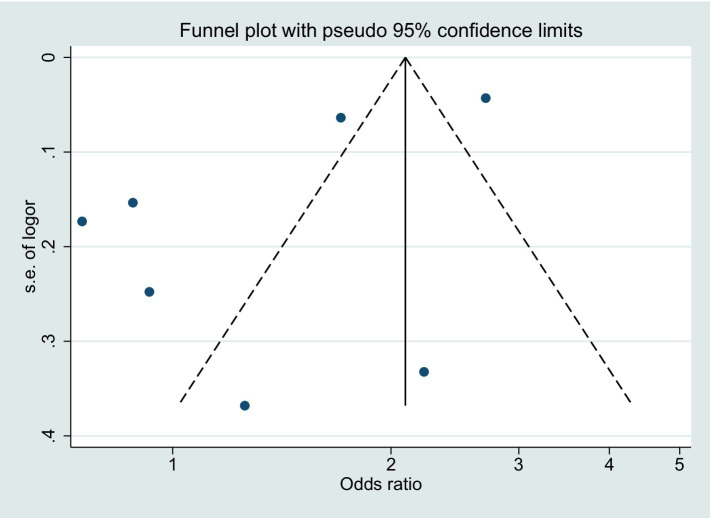
Funnel plot displaying pseudo 95% confidence limits. SE, standard error; OR, odds ratio.

Regarding publication bias, the funnel plot for BPD risk demonstrated some degree of asymmetry (Figure 4). However, considering that the point falls outside the dotted line, it is likely that the asymmetry is a result of inter-study heterogeneity.

### Subgroup analysis

**Figure 5:**
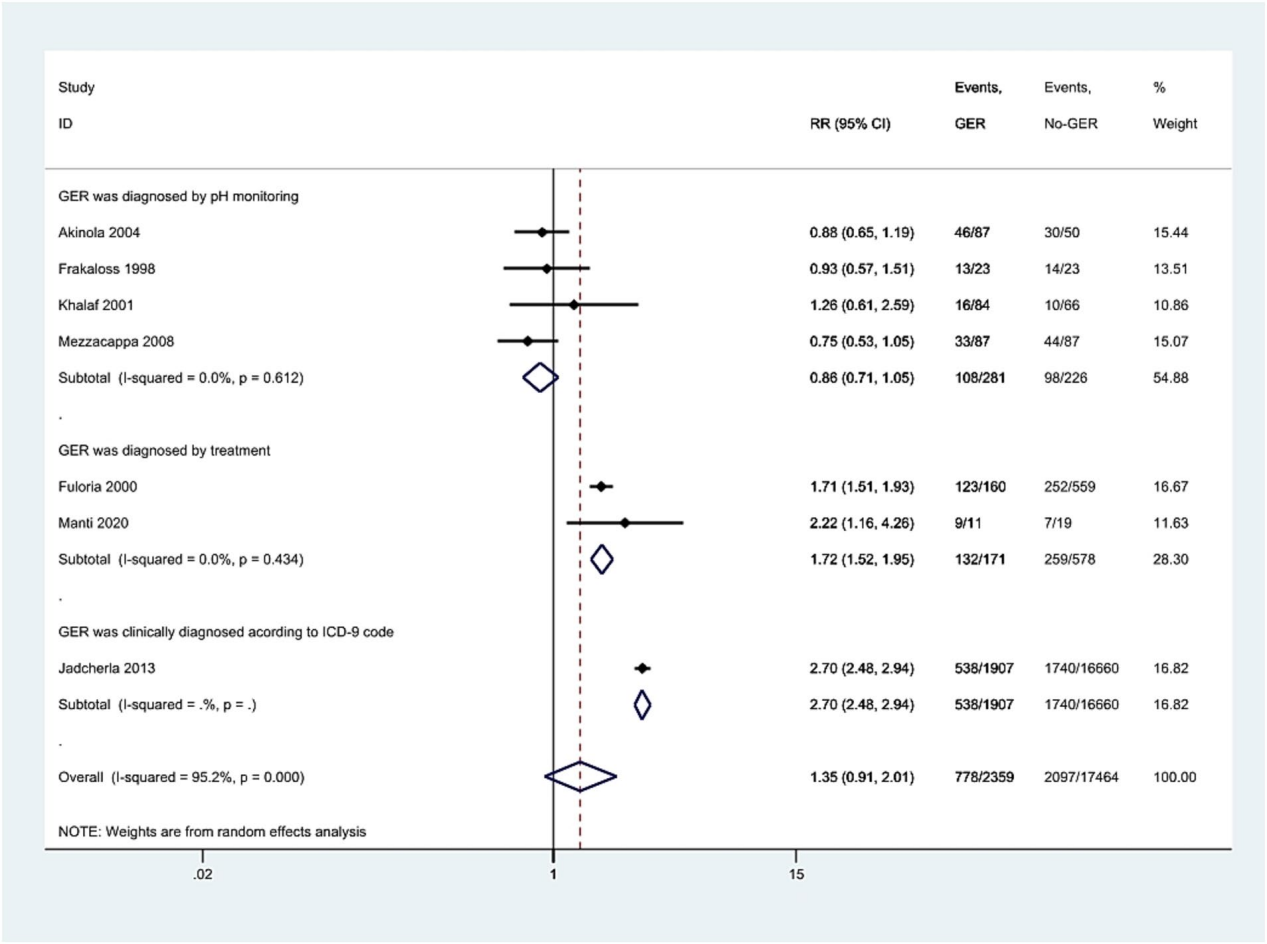
Meta-analysis exploring the association of GER diagnosed through pH monitoring, treatment, or ICD-9, respectively, with BPD in preterm infants. The results present both individual and pooled RR values along with 95% CIs. GER, gastroesophageal reflux; BPD, bronchopulmonary dysplasia; ICD, International Classification of Diseases; RR, risk ratio; CI, confidence interval.

We divided the study population into three subgroups based on the diagnostic criteria for GER exposure: GER diagnosed by pH monitoring, GER diagnosed by treatment, and GER clinically diagnosed according to ICD-9 codes. The *I*^2^ values for the first two subgroups were 0.0, 0.0% with *p*-values of 0.612 and 0.434, respectively. The corresponding RR values and 95% CIs for the two groups were 0.86 (0.71, 1.05) and 1.72 (1.52, 1.95). Notably, there was only one study in which clinically diagnosed GER based on ICD codes showed a significant correlation with BPD (RR = 2.70, 95% CI = 2.48, 2.94, Figure 5).

## Discussion

Our meta-analysis, which combined outcomes from 19,823 infants across seven individual studies, revealed significant heterogeneity among the studies. While some heterogeneity may be attributed to small sample size effects, the primary source of variability stemmed from differences in the diagnostic criteria used for GER (GERD). Subgroup analysis provided valuable insights, indicating that GER (GERD) diagnosed by pH monitoring was not associated with BPD, aligning with findings by Sindel et al. (19). However, notably, GER (GERD) diagnosed based on clinical therapy or ICD-9 codes showed a significant association with BPD, as indicated by a higher RR value that was both statistically significant and greater than that of non-GER infants. This finding suggests that preterm infants diagnosed with GER based on clinical symptoms and therapy, may be at an increased risk of developing BPD. Clinical diagnosis likely captures more severe cases of GER, which could potentially have a greater impact on lung function and contribute to the pathogenesis of BPD. To the best of our knowledge, this is the first meta-analysis to assess the correlation between GER and BPD in preterm infants.

Currently, the diagnosis of GER (GERD) in infants is primarily based on clinical history and physical examination. However, in the absence of a definitive diagnostic gold standard, existing studies have utilized esophageal pH monitoring with reflux index (RI) cutoff values (varying from 5 to 10%) for establishing GER diagnosis (Table 1). The RI is defined as the percentage of total monitoring time during which the distal esophageal pH remains below 4.0. The frequent feeding in neonates consistently dilutes gastric pH, resulting in approximately 24.5% of time with gastric pH < 4, varying from 0.6 to 69.1% (28). Traditional pH monitoring has considerable diagnostic variability and cannot detect non-acid or alkaline reflux events. These substantial technical limitations introduce considerable diagnostic uncertainty, thereby potentially confounding any observed association between GER and BPD.

Approximately 70–85% of infants experience reflux during the first 2 months of life (3). The combined pH-multichannel intraluminal impedance (pH-MII) monitoring technique overcomes the limitations of conventional 24-h pH monitoring by providing comprehensive data on both the frequency and chemical composition of reflux episodes. It was reported that during 704.3 h of recordings, only 54% of GER episodes were associated with symptoms (29). Symptoms of GER include recurrent vomiting, regurgitation, apnea, bradycardia or tachycardia, decreased oxygen saturation, irritability, abnormal neck posturing, excessive crying, swallowing and feeding difficulties, wheezing, or coughing (30). A prospective observational cohort study involving 46 preterm infants with GER-related symptoms found that infants with BPD had an increased number of reflux events, which were more frequently associated with symptoms (16). The differing results between clinically diagnosed GER and laboratory-based diagnosis highlight the challenges in accurately identifying and characterizing GER in preterm infants.

Research on the mechanisms of BPD development in preterm infants with GER is relatively limited. The concentration of pepsin was found to be increased in the tracheal aspirate of preterm infants with BPD (7). Inhalation of stomach contents can lead to chemical and biological damage to the lungs and reduce the lungs’ bacterial clearance capacity, potentially contributing to bacterial or ventilator-associated pneumonia (7, 31). Animal experiments also found that the particulate matter in stomach contents increased the production of lung inflammatory cells and released inflammatory factors, thus mediating lung injury (32). Additionally, infants with BPD may be at increased risk for GER due to dyspnea and transient elevated intra-abdominal pressure associated with coughing, crying, and airflow obstruction, which reduces LES tension and increases the occurrence of transient lower esophageal sphincter relaxation (19). Fuloria et al. (24) postulated that pharmacologic interventions for apnea may reduce LES tone. Additionally, pulmonary hyperinflation could displace the LES into the thoracic cavity, while increased intra-abdominal pressure during expiration might further elevate the likelihood of GER in infants with BPD. It is possible that these reflux episodes reach a threshold of severity to significantly influence lung injury or that other factors, such as the underlying lung immaturity or inflammation associated with BPD, play a dominant role in disease progression.

Several limitations of this meta-analysis need to be carefully considered. First, all studies were retrospective, which makes them more susceptible to observational biases. Neither the temporal relationship nor causality between GER and BPD can be conclusively established. Then, only two publications (10, 24) of the included studies specifically examined the direct association between GER and BPD. The remaining four studies (6, 23, 25–27) evaluated BPD as one of multiple potential clinical correlates of GER, rather than as a primary focus of investigation. Furthermore, publication bias cannot be excluded since the studies were conducted only in the English language. Finally, heterogeneity is a significant issue affecting the interpretation of meta-analysis results. The presence of heterogeneity may result from small sample bias and diagnostic bias. Although there were differences in the diagnostic criteria and methods for GER among the studies included in this review, subgroup analysis helped mitigate heterogeneity in the results of this study.

Despite the included studies showing no evident differences in gestational age, weight, and other factors in the study population, the influence of mechanical ventilation, caffeine, and other confounders on the occurrence of BPD was not entirely excluded. Only one study accurately assessed and controlled for confounding factors between infants with and without GER. Future studies should establish clear diagnostic criteria for the GER population and include larger sample sizes to provide more robust conclusions.

In summary, this meta-analysis demonstrated that GER diagnosed by pH monitoring had no potential role in the development of BPD. However, GER requiring therapy or clinically diagnosed based on ICD-9 codes might be a risk factor for BPD. Future studies with larger sample sizes and standardized diagnostic criteria are warranted to elucidate the precise mechanisms underlying the association between GER and BPD in preterm infants. These investigations should also consider potential confounders and explore the impact of specific treatment strategies targeting GER on the incidence and severity of BPD. A better understanding of the interplay between GER and BPD will have important implications for clinical management and the development of preventive interventions in this vulnerable population.

## Data Availability

The datasets presented in this study can be found in online repositories. The names of the repository/repositories and accession number(s) can be found in the article/Supplementary material.
