# Supplementary material for: Association between gastroesophageal reflux and bronchopulmonary dysplasia in preterm infants: a systematic review and meta-analysis
Source: Front Nutr. 2025 Jun 24;12:1562939. doi: 10.3389/fnut.2025.1562939 (PMC12234343; doi:10.3389/fnut.2025.1562939)
Supplement: Supplementary file 1 [file Data_Sheet_1.docx]

**Supplementary Online Content**

**Supplementary Table 1.** Literature search strategy

| **Database: Medline (-Oct 19, 2024)** | | |
| --- | --- | --- |
| **#** | **Searches** | **Results** |
| 1 | Premature Birth | 68,644 |
| 2 | Infant, Premature/ | 113,098 |
| 3 | Obstetric Labor, Premature/ | 37,475 |
| 4 | Infant, Newborn/ | 695,704 |
| 5 | preterm or pre-term or prematur* or term | 1,714,559 |
| 6 | lbw | 5,368 |
| 7 | low birth weight | 68,158 |
| 8 | vlbw | 4,536 |
| 9 | very low birth weight | 61,884 |
| 10 | extra low birth weight | 341 |
| 11 | (((((((((Premature Birth) OR (Infant, Premature/)) OR (Obstetric Labor, Premature/)) OR (Infant, Newborn/)) OR (preterm or pre-term or prematur* or term)) OR (lbw)) OR (low birth weight)) OR (vlbw)) OR (very low birth weight)) OR (extra low birth weight) | 2,250,056 |
| 12 | reflux | 74,229 |
| 13 | Gastroesophageal reflux | 38,783 |
| 14 | GER | 12,023 |
| 15 | Gastro-oesophageal reflux | 40,137 |
| 16 | reflux index | 7,348 |
| 17 | duodenal gastric reflux | 2,722 |
| 20 | (((((reflux) OR (Gastroesophageal reflux)) OR (GER)) OR (Gastro-oesophageal reflux)) OR (reflux index)) OR (duodenal gastric reflux) | 84,251 |
| 21 | bronchopulmonary dysplasia | 10,718 |
| 22 | chronic lung disease | 185,911 |
| 23 | BPD | 13,909 |
| 24 | CLD | 5,874 |
| 25 | (((bronchopulmonary dysplasia) OR (chronic lung disease)) OR (BPD)) OR (CLD) | 208,504 |
| 26 | (((((((((((Premature Birth) OR (Infant, Premature/)) OR (Obstetric Labor, Premature/)) OR (Infant, Newborn/)) OR (preterm or pre-term or prematur* or term)) OR (lbw)) OR (low birth weight)) OR (vlbw)) OR (very low birth weight)) OR (extra low birth weight)) AND ((((((reflux) OR (Gastroesophageal reflux)) OR (GER)) OR (Gastro-oesophageal reflux)) OR (reflux index)) OR (duodenal gastric reflux))) AND ((((bronchopulmonary dysplasia) OR (chronic lung disease)) OR (BPD)) OR (CLD)) | 266 |

| **Database: Embase (on OVID platform)**  **(-Oct 19, 2024)** | | |
| --- | --- | --- |
| **#** | **Searches** | **Results** |
| 1 | Premature Birth | 12033 |
| 2 | Infant, Premature/ | 126731 |
| 3 | Obstetric Labor, Premature/ | 31278 |
| 4 | Infant, Premature, Diseases/ | 80427 |
| 5 | Infant, Newborn/ | 559934 |
| 6 | preterm.mp. | 137873 |
| 7 | low birth weight.mp. | 71222 |
| 8 | Infant, Low Birth Weight/ | 40965 |
| 9 | very low birth weight.mp. | 17721 |
| 10 | Infant, Extremely Low Birth Weight/ | 4369 |
| 11 | lbw.mp. | 7409 |
| 12 | vlbw.mp. | 6314 |
| 13 | or/1-11 | 723288 |
| 14 | reflux.mp. | 132629 |
| 15 | Gastroesophageal reflux.mp. | 74841 |
| 16 | GER.mp. | 5577 |
| 17 | Gastro-oesophageal reflux.mp. | 6229 |
| 18 | reflux index.tw. | 501 |
| 19 | duodenal gastric reflux.tw. | 28 |
| 20 | or/14-19 | 134832 |
| 21 | bronchopulmonary dysplasia.tw. | 12658 |
| 22 | chronic lung disease.tw. | 12095 |
| 23 | BPD.tw. | 18339 |
| 24 | CLD.tw. | 7934 |
| 25 | or/21-24 | 41581 |
| 26 | 13 and 20 and 25 | 139 |

| **Database:CENTRAL (Cochrane Central Register of Controlled Trials)**  **(-Oct 19, 2024)** | | |
| --- | --- | --- |
| **#** | **Searches** | **Results** |
| 1 | infant, premature | 10773 |
| 2 | Premature Birth | 9814 |
| 3 | Infant, Newborn | 28270 |
| 4 | Infant, Premature, Diseases | 2510 |
| 5 | preterm.mp. | 22 |
| 6 | low birth weight.mp. | 8 |
| 7 | Infant, Low Birth Weight | 6127 |
| 8 | very low birth weight.mp. | 8 |
| 9 | Infant, Extremely Low Birth Weight | 915 |
| 10 | lbw.mp. | 31 |
| 11 | #1 or #2 or #3 or #4 or #5 or #6 or #7 or #8 or #9 or #10 | 34342 |
| 12 | reflux.mp. | 2 |
| 13 | Gastroesophageal reflux | 5142 |
| 14 | Gastro-oesophageal reflux | 964 |
| 15 | reflux index.tw. | 0 |
| 16 | GER.mp. | 0 |
| 17 | duodenal gastric reflux.mp. | 0 |
| 18 | #12 or #13 or #14 or #15 or #16 or #17 | 5455 |
| 19 | bronchopulmonary dysplasia | 2118 |
| 20 | chronic lung disease | 19592 |
| 21 | BPD. mp. | 74 |
| 22 | CLD.mp. | 0 |
| 23 | #19 or #20 or #21 or #22 | 21348 |
| 24 | #11 and #18 and #23 | 27 |

**Supplementary Table 2.** Inclusion and Exclusion Criteria for a Systematic Review and Meta-analysis of BPD Outcomes Associated with infants Exposure to GER•

| **Study (country)**  **[Risk of bias**  **assessment]** | **Inclusion criteria** | **Exclusion criteria** |
| --- | --- | --- |
| **A exposure of GER versus non-GER** | | |
| Akinola, 2004 (U.S.)^10^  ★★★★★★★ | - GA <32 weeks - had clinical symptoms suggestive of GER - had documented results from extended esophageal pH monitoring   [GER group, n=87; non-GER group, n=50]  o GER was diagnosed by pH monitoring RI ≥ 10 | - major congenital anomalies known to be associated with GER |
| Frakaloss, 1998(U.S.) ^23^  ★★★★★★★ | - Premature infants (gestational age less than 37 weeks at birth) with clinically significant GER [GER group, n= 23; non-GER group, n=23]   o The diagnosis of GER was confirmed by pH probe(RI >5), milk scan, and barium contrast study | - conditions with the potential to affect growth, including gastrointestinal tract anomalies, severe neurologic disease, surgically treated necrotizing enterocolitis, chromosomal abnormalities, or malformations impairing normal feeding |
| Fuloria, 2000(U.S.)^3^  ★★★★★★★ | - all VLBW (˂1500 g) infants with CLD [GER group, n=160; non-GER group, n=559]   o GER was diagnosed by tests or treatment for GER | - NA |
| Jadcherla, 2013 (U.S.)^6^  ★★★★★★★★ | - GA 22 to 36 weeks of 33 hospitals [GER group, n=1907; non-GER group, n=16660]   o GER was clinically diagnosed acording to ICD-9 code | - term infants - < 22 weeks’ GA or birth weight < 400 g - data for age missing at admission - age at admission > 30 d - missing the PHIS acuity score |
| Khalaf, 2001(U.S.) ^25^  ★★★★★★★ | - 150 infants (GA, 23–42 weeks, birth weight,470–4700 g) who had a five-channel study with a pH recording to evaluate for GER. [GER group, n=84; non-GER group, n=66]   o GER was diagnosed if RI ≥ 6 | - NA |
| Manti, 2020(Italy) ^26^  ★★★★★★★ | - 30 pre-school children (age range 3-6 years) who were born before 32 weeks of gestational age and affected by BPD[GER group, n=11; non-GER group, n=19]   o GER was defined as reflux with pathologic consequences requiring any kind of antireflux therapy | - pulmonary, cardiac congenital malformations or any other pathological condition that did not allow a correct execution of the IOS technique |
| Mezzacappa 2008 (U.S.) ^27^  ★★★★★★★★ | - All preterm infants (birthweight <2000 grams and gestational age ≤37 weeks) who had undergone prolonged distal intra-esophageal pH monitoring. [GER group, n=87; non-GER group, n=87]   o GER was diagnosed if RI ≥ 10 | - non-standardized monitoring conditions - technical problems were encountered |

**Legend:**

VLBW – very low birth weight; g – grams; GA – gestational age; NS – not stated/defined; ICD – International Classifi cation of Diseases, Ninth Revision; GER - gastroesophageal reflux.

Newcastle-Ottawa Scale: ★ – point awarded; ½ – half point awarded

**Supplementary Table 3.** Newcastle-Ottawa Scale Quality Assessment Scores for Non-Randomized Studies Included in a Systematic Review and Meta-analysis of BPD Outcomes Associated with Preterm Exposure to GER

| **Study, Year (Country)** | | **Total score (max: 9**★**)** | **Component scores** | | | | | | | |
| --- | --- | --- | --- | --- | --- | --- | --- | --- | --- | --- |
|  |  |  | Representativeness of exposed  cohort  (max: ★ ) | Selection of nonexposed  cohort  (max: ★ ) | Ascertainment of  exposure (max:  ★ ) | Demonstration that outcome of  interest was not present at study  start (max: ★ ) | Comparability of cohorts based on  the design or  analysis*  (max: ★★) | Ascertainment of  outcome (max: ★ ) | Follow-up long  enough for outcomes to  occur? (max: ★ ) | Adequacy of  cohort follow up  (max: ★ ) |
| *GER was diagnosed by PH monitoring* | | | | | | | | | | |
| Akinola, 2004 (U.S.)^10^ | 7 ★★★★★★★ | | ★ | ★ | ★ | ☆ | ★☆ | ★ | ★ | ★ |
| Frakaloss, 1998 (U.S.) ^23^ | 7 ★★★★★★★ | | ★ | ★ | ★ | ☆ | ★☆ | ★ | ★ | ★ |
| Khalaf, 2001(U.S.) ^25^ | 7 ★★★★★★★ | | ★ | ★ | ★ | ☆ | ★☆ | ★ | ★ | ★ |
| Mezzacappa 2008 (U.S.) ^27^ | 8 ★★★★★★★★ | | ★ | ★ | ★ | ☆ | ★★ | ★ | ★ | ★ |
| *GER was clinically diagnosed* | | | | | | | | | | |
| Fuloria, 2000(U.S.) ^24^ | 7 ★★★★★★★ | | ★ | ★ | ★ | ☆ | ★☆ | ★ | ★ | ★ |
| Jadcherla, 2013 (U.S.)^6^ | 8 ★★★★★★★★ | | ★ | ★ | ★ | ☆ | ★★ | ★ | ★ | ★ |
| Manti, 2020(Italy) ^26^ | 7 ★★★★★★★ | | ★ | ★ | ★ | ☆ | ★☆ | ★ | ★ | ★ |

**Legend:**

★ – point awarded; ☆ – no point awarded, max – maximum.
